# Dynamics of dopamine transients in human olfactory epithelium during reward learning

**DOI:** 10.64898/2026.10.07.757414

**Published:** 2026-10-14

**Authors:** L. Paul Sands, Seth R. Batten, Alec E. Hartle, Terry Lohrenz, Jason White, Leonard S. Barbosa, W. Matt Howe, Dan Bang, Peter Dayan, Qiaohan Yang, Adam Dede, Andrew Sheriff, Gregory Lane, Bruce K. Tan, Paul Lenkowski, Christina Zelano, P. Read Montague

## Abstract

Dopamine signaling is crucial for motivation, movement, and learning, but has remained beyond the reach of measurement in healthy humans. Here, we measured sub-second dopamine in the olfactory epithelium of healthy humans during two reward learning tasks with distinct reward types: liquid reward during a conditioning task and monetary reward during an economic exchange game. In both tasks, as trials progress, peak dopamine transients shift from rewarding outcomes back to reward-predictive cues. In the economic game, this pattern retrains quickly when reward magnitude or reward timing is changed without warning. These findings match the time transfer characteristics and amplitude dependence of temporal difference prediction error signals. The results also establish a new, minimally invasive route for recording fast dopamine signaling in healthy humans: the nose.

